# Supplementary material for: Two’s company, three species is a crowd? A webcam-based study of the behavioural effects of mixed-species groupings in the wild and in the zoo
Source: PLoS One. 2023 Apr 20;18(4):e0284221. doi: 10.1371/journal.pone.0284221 (PMC10118120; doi:10.1371/journal.pone.0284221)
Supplement: S1 File — (DOCX) [file pone.0284221.s001.docx]

Supplementary information

Appendix A

| **Country** | **Camera Location** | **Camera Name** | **Number of Observations** | **Web links for (1) camera and (2) weather (correct as of March 2023)** |
| --- | --- | --- | --- | --- |
| South Africa | *Balule Nature Reserve* | Naledi Dam  30.88564° S, 24.20735° E | 5 | (1) <https://www.africam.com/wildlife/stream/naledi-dam>  (2) <https://weather.com/en-GB/weather/today/l/-24.27,30.90?par=google> |
|  | *Pilanesberg National Park* | Kwa Maritane  27.14565° S, 25.33209° E | 16 | (1) <https://www.africam.com/wildlife/stream/kwa-maritane>  (2) <https://weather.com/en-GB/weather/today/l/-25.33,27.15?par=google> |
|  | *Tembe Elephant Park* | Tembe Elephant Park  27.0486° S, 32.4224° E | 95 | (1) <https://explore.org/livecams/african-wildlife/tembe-elephant-park>  (2) <https://weather.com/en-GB/weather/today/l/-27.05,32.42?par=google> |
|  | *Madikwe National Park* | Tau Waterhole  26.20544° S, 24.70795° E | 45 | (1) <https://explore.org/livecams/african-wildlife/tau>  (2) <https://weather.com/en-GB/weather/today/l/-24.76,26.28?par=google> |
|  | *Olifants West Game Reserve* | Olifants River  30.87158° S, 24.17644° E. | 53 | (1) <https://explore.org/livecams/africam/olifants-river>  (2) <https://weather.com/en-GB/weather/today/l/-24.15,31.04?par=google> |
|  |  | The Naledi Cat Eye  30.9703° S, 25.4745° E | 8 | (1) <https://explore.org/livecams/africam/naledi-cat-eye>  (2) <https://weather.com/en-GB/weather/today/l/-24.15,31.04?par=google> |
|  | *Sabi Sands Game Reserve* | Nkorho Bush Lodge  24.7339° S, 31.5971° E | 25 | (1) <https://explore.org/livecams/african-wildlife/nkorho-bush-lodge>  (2) <https://weather.com/en-GB/weather/today/l/-24.93,31.52?par=google> |
| Kenya | *Laikipia County, Mpala Research Centre* | African Animals - Watering Hole  0.2924° N,  36.8985° E | 8 | (1) <https://explore.org/livecams/african-wildlife/african-watering-hole-animal-camera>  (2) <https://weather.com/en-GB/weather/today/l/0.36,36.78?par=google> |
|  | *Mpala Research Centre* | African Animals (river) | 12 | (1) <https://explore.org/livecams/african-wildlife/african-animal-lookout-camera>  (2) <https://weather.com/en-GB/weather/today/l/0.36,36.78?par=google> |
|  | *Mpala Research Centre* | African River Wildlife (upriver view) | 6 | (1) <https://explore.org/livecams/african-wildlife/african-river-wildlife-camera>  (2) <https://weather.com/en-GB/weather/today/l/0.36,36.78?par=google> |
| United States | *San Diego Zoo Safari Park* | Giraffe Cam  33.1017° N, 117.0013° W | 93 | (1) <https://sdzsafaripark.org/cams/giraffe-cam>  (2) <https://weather.com/en-GB/weather/today/l/32.74,-117.15?par=google> |

Appendix B

| **Species** | **No. of focal obs** | **Percentage of focal obs from wild data (%)** | **Frequency of mixed-species groupings (%)** | **Vigilance rate in single-species groups** | **Vigilance rate in mixed-species groups** |
| --- | --- | --- | --- | --- | --- |
| Impala (*Aepyceros melampus)* | 57 | 94.7 | 57.9 | 0.64 | 0.45 |
| Giraffe (*Giraffa camelopardalis)* | 37 | 48.6 | 37.9 | 0.63 | 0.26 |
| Nyala (*Tragelaphus angasii)* | 32 | 100 | 40.5 | 1.01 | 0.68 |
| Waterbuck (*Kobus ellipsiprymnus)* | 29 | 69.0 | 72.4 | 0.64 | 1.13 |
| White Rhinoceros (*Ceratotherium simum)* | 24 | 0 | 41.7 | 0.40 | 0.35 |
| Blue Wildbeest (*Connochaetes taurinus)* | 23 | 69.6 | 47.8 | 0.36 | 0.43 |
| Plains Zebra (*Equus quagga*) | 22 | 100 | 77.3 | 0.70 | 0.45 |
| Egyptian (*Alopochen aegyptiaca)* | 20 | 100 | 45 | 0.85 | 1.60 |
| Warthog (*Phacochoerus africanus)* | 16 | 100 | 37.5 | 0.67 | 0.48 |
| African elephant (L*oxodonta africana)* | 15 | 100 | 60 | 0.28 | 0.75 |
| Lechwe (*Kobus leche)* | 13 | 0 | 84.6 | 0.34 | 0.25 |
| Cape Bushbuck (*Tragelaphus sylvaticus)* | 12 | 100 | 16.7 | 0.77 | 1.14 |
| Stork (*Ciconia episcopus)* | 10 | 100 | 50 | 2.23 | 1.37 |
| Fringe-eared Oryx (*Oryx beisa callotis)* | 10 | 0 | 70 | 0.18 | 0.33 |
| Cape Buffalo (*Syncerus caffer)* | 9 | 11.1 | 77.8 | 0.42 | 0.30 |
| Greater Kudu (*Tragelaphus strepsiceros )* | 9 | 100 | 55.6 | 0.72 | 0.89 |
| Vervet (*Chlorocebus pygerythrus)* | 6 | 100 | 0 | 0.35 | n/a |
| Chacma Baboon (*Papio ursinus)* | 5 | 100 | 20 | 0.47 | 0.57 |
| Common Ostrich (*Struthio camelus)* | 3 | 100 | 100 | n/a | 0.77 |
| Grey heron (*Ardea cinerea)* | 2 | 100 | 100 | n/a | 0.56 |
| White-faced whistling duck (*Dendrocygna viduata)* | 2 | 100 | 50 | 1.61 | 0.73 |
| Hamerkop (*Scopus umbretta)* | 2 | 100 | 100 | n/a | 1.33 |
| Lion *Panthera leo* | 2 | 100 | 50 | 0.21 | 0.10 |
| Spur-winged goose *Plectropterus gambensis* | 1 | 100 | 100 | n/a | 0.35 |
| Grey Rhebok *Pelea capreolus* | 1 | 100 | 0 | 1.80 | n/a |
| Sitatunga *Tragelaphus spekii* | 1 | 100 | 100 | n/a | 2.75 |
| Common Eland *Taurotragus oryx* | 1 | 100 | 100 | n/a | 0.14 |
| Hartebeest *Alcelaphus buselaphus* | 1 | 100 | 100 | n/a | 1.12 |
| Kirk's dik-dik *Madoqua kirkii* | 1 | 100 | 0 | 0.87 | n/a |
