# Supplementary material for: Two’s company, three species is a crowd? A webcam-based study of the behavioural effects of mixed-species groupings in the wild and in the zoo
Source: PLoS One. 2023 Apr 20;18(4):e0284221. doi: 10.1371/journal.pone.0284221 (PMC10118120; doi:10.1371/journal.pone.0284221)
Supplement: Attachment — Submitted filename: replies to editor and reviewers.docx [file pone.0284221.s003.docx]

**Replies to editor:**

Dear authors,

Since it has been hard to find potential reviewers for the ms I did myself reviewed it. I think that is an original idea and method to survey and compare behaviours between wildlife and captive animals. However, I agree with the referee that this paper, in its current setup, may not give much information that is useful within zoos. I also agree with the referee that the discussion is over-reached – e.g. how this information can be used directly in terms of managing zoos? In addition, it is hard to compare the behaviour of wild animal, with captive ones, the latest have less predation risk, food availability, no harsh weather conditions, within others… I suggest authors to erect alternative hypotheses to their main conclusion, i.e. “The results suggest that these species benefit from increased perceived safety in larger groups, regardless of the species making up that group”, accounting for the main differences between wild and captive species behaviour. As rose by the referee, the way the data is presented in terms of combined zoo and wild data may be masking lots of differences, and it may be an error to join them together (due to the differences between the environments but principally the fact that zoos are keeping animals in pre-defined groups, these associations aren’t a choice). I would also encourage the authors to re-do the analyses with the two groups (zoo vs wild) as separate entities, and then compare them descriptively in the discussion. I would also encourage authors to look at fuller activity budgets rather than just vigilance. Authors should also state much clearly on the rationale for solely including vigilance since its application in zoos is relatively limited, especially as high levels of vigilance in the zoo environment it is not desirable. And finally, they should state much clearly, how their research may be useful or may be applied in zoos.

**Replies to reviewer 1**

Reviewer #1: Dear authors,

Thank you for submitting this paper, I thought it was an interesting piece of work and is quite a nice idea in terms of understanding a little more about mixed species associations in the wild and considering how that can be transferred over to zoos.

I did find some aspects of the work a little confusing and I think it needs some work to make the findings clearer and more applicable. My main comment is that I think you need to separate out the zoo and wild data for analysis. These two environments are so completely different that I think by combining them you are muddying the waters and I don’t think the conclusions hold valid because of it. The zoo is of course an enforced mixed species exhibit and an exhibit with no natural predators so is a completely different environment to the wild, and I think that is why they need to be treated separately. I am also unclear why you chose to focus only on vigilance, when you collected a whole activity budget. Vigilance is something we really drive hard not to have excessive amounts of in a zoo, and I think comparing whole activity budgets would be much more beneficial in terms of the aims of your study.

**I have now separated out the analysis for zoo and wild data, and I have also included further information on a wider variety of behaviour types.**

General comments on methods:

More detail needed on the zoo enclosure – approx. how big is the space and how much of that space was covered by the camera.

**I have added more information on the zoo enclosure.**

Habitat type is confusing, is this wild animals only? How are you defining different types of habitat within the zoo?

**It is used for both the zoo and the wild individuals and I have clarified this in the text and explained why it was also used in the zoo.**

L263/264: why did you use chi squared here? This data doesn’t appear to be categorical so a t-test or mann whitney would have been more appropriate

**I have re-phrased this paragraph to make more sense as a chi-squared test.**

Your factors in relation to ‘predicted likelihood of forming mixed social grps’ don’t make sense for zoos – the grps are formed by the zoo, there is nothing to predict it. you can look at whether they predict proximity but that is slightly different to what I think you are suggesting

**In the study I labelled a 'group' as being within six body lengths of each other, to differentiate animals that are just in the same area from animals that are potentially associating. I explained this in the methods but have also now clarified this further now so that it hopefully makes more sense. It is essentially about predicting proximity, as you suggest, as in the study my definition of a group is purely based on the extent of proximity between individuals. For example, whilst white white rhinoceroses and Thompson’s gazelles are both in the same enclosure, they were never observed to be anywhere near six body lengths from each other, and so were never labelled as being in the same group.**

I don’t think within 6 body lengths is appropriate for a zoo setting – that’s a pretty large distance from another animal, I think you need a strong rationale for why you chose that and not for example within one or two body lengths as would normally be done.

**I hope that my further detail on the size of the enclosure might help to explain why we felt that six body lengths was appropriate.**

You need details on number of grps where you did know the sexes of animals as the number of these will show whether your results in relation to this are affected by small samples.

**I have added information in the methods section and results.**

Information needed on the zoo in terms of numbers of species/diversity of sp in the mixed sp enclosure.

**I have added this information in the methods.**

General comments on discussion:

Discussion feels a bit muddled and like it has lost its way a little bit – I think you need to split out zoo and wild data and then consider more what we can do with the wild data to help support zoo management decisions. I also think the discussion will be stronger if you look at activity budgets more fully.

**These changes have been made and the discussion is now more fluid.**

Line by line comments:

L40: give examples of habitat type.

**I have provided examples.**

L47/48: I would argue that increased vigilance is not something.

**I have removed this section.**

L85: ‘have much potential’ is a bit clunky – suggest rewording.

**I have re-worded this sentence.**

L88: maybe, you can’t guarantee these risks can be avoided.

**I have re-worded to highlight this.**

L92: enclosures.

**Resolved.**

L95: whether these associations change and if they influence… - you don’t know they definitely do – also, is this referring to zoo or wild? Needs clarity throughout on the two environments.

**I have clarified this.**

L108/109: typos.

**Resolved.**

L113: provide some examples of sp which are commonly kept in mixed sp grpings

**I have added a relevant citation.**

L115: in the wild? Zoo?

**Re-phrased.**

L124: not sure how you are answering Q 3?

**I have re-worded this question to make more sense with the analysis done.**

L125: Q #4- you are forcing the sp associations within a zoo so I think this needs to refer to spatial associations.

**I felt that the location of the cameras near water sources in the wild locations created a somewhat similar situation to within the zoo enclosure, where animals are to some extent forced to use the same space (in the zoo because they are in an enclosure, and in the wild because they are all using the same resource), however I aimed to use the six body lengths to identify species that were closer than required even whilst using the same resource, both at the zoo and in the wild spaces.**

L130: what do you mean by ‘particularly close proximity’?

**I have clarified what is meant here by referring directly to the six body lengths measurement used.**

L134: six body lengths is huge in a zoo – needs strong rationale

**I have added details of the size of this San Diego Zoo enclosure. It is a large space, roughly 12 hectares in size, within which it is very easy for animals to stay at a significant distance to each other. Many species in this enclosure were never observed within six body lengths of each other.**

L134: if animals are forced to associate because of resources how do you know it is a choice to aggregate how they are?

**Please see response to comment L125.**

L149: you were only looking at vigilance so hypothesis #5 feels the opposite of hypothesis #4

**I have now included analyses of additional behaviours rather than just looking at vigilance.**

**H4 says that overall vigilance will be lower, but doesn’t suggest an impact of grouping, whereas H5 is focussing on the impact of grouping, and saying that vigilance may be even lower when in a mixed species group in captivity.**

L252: it is not clear if this is wild animals only, although I am not clear how it fits with the zoo environment, needs to specify

**Please see response to comment L134. The zoo enclosure is a very large space with a waterhole, areas of vegetative cover, and areas of open grass.**

L308: see comments above – I don’t think it makes sense to combine the data.

**I have changed the analyses to look at zoo and wild data separately.**

L310: not sure what is meant by ‘this was not significant for individual sp either’?

**I have re-worded this to be clearer.**

L313: is this wild or zoo? need to clarify

**Re-phrased.**

Tbl 5: make subtitles in the table bold so it’s clearer

**Done.**

L380: not sure what the n=150 refers to?

**I have clarified this.**

L381: you don’t know it is significantly influencing it – it stands to reason that larger grps will consist of more species but that’s not to say that correlation = causation here. Be mindful of this in interpretation through the discussion too.

**I have removed focal group size from the analysis as I realise it wasn't suitable.**

L397: how does this relate to zoos?

**Please see response to comment L134 & L252.**

L422 – 424: not sure how this has been reached from the data presented?

**Line deleted.**

L450: reduced vigilance as compared to what? Giraffe on their own? Or comparison to the wild?

**I have clarified this statement.**

L458 – 461: it’s quite likely predators were just off camera. Predators in the wild are driving associations (amongst other things) so they’re a big driver of social grps

**I have now removed the comment about the presence of predators not significantly influencing the associations and have made a comment about this limitation in the limitations section.**

L464 – 468: you haven’t included this information in your results – but I do think you need to

**I have now included additional information about general activity other than vigilance.**

L469 – 471: Yes! Which is what makes this study quite confusing

**I have tried to explain the way I was looking at associations and mixed species groupings more clearly, please see above my comments on six body lengths and this distinction of a group.**

L481: this is a strong rationale for analysing this data separately and only comparing where you have similar mixes between wild and zoo environments

**I have done this now.**

L528/529: they surely wouldn’t do it if it did create stress. There could be multiple other environmental reasons causing this.

**Noted.**

L530: how did sp diversity compare in zoo/wild?

**I have added information on this in the results and discussion.**

L537: why do they perform more vigilance?

**I have added an explanation for this.**

L550: needs a citation – not sure how/why this is considered more energetically costly than vigilance as a state behaviour?

**Comment removed after change to analyses.**

L576: how many grps are sexually dimorphic? How representative is this?

**I have added information on this in the methods and results.**

L583: how does this compare with data from grps of ungulates

**Re-phrased.**

L635: you could look at this with your data – and I would encourage you to look at this with your data.

**I didn't collect data on inter-species interactions, only intra-species interactions, as there was already a lot of data to collect and I found it easier to only have to focus on the behaviour of the focal species.**

L649: you only looked at vigilance

**We now include other behaviours.**

L654 – 656: I don’t think you can say this as you’ve not really got any idea why vigilance reduced

**Removed.**
