# Supplementary material for: Two’s company, three species is a crowd? A webcam-based study of the behavioural effects of mixed-species groupings in the wild and in the zoo
Source: PLoS One. 2023 Apr 20;18(4):e0284221. doi: 10.1371/journal.pone.0284221 (PMC10118120; doi:10.1371/journal.pone.0284221)
Supplement: Attachment — Submitted filename: PLoS One replies to reviewers.docx [file pone.0284221.s004.docx]

PLoS One replies to reviewers

Editor comment

I have received now the comment from the reviewer and I am agree that the Ms has improved. I will recommend to you to follow all suggestions rose by reviewer, and pay special attention to the assertion left in the discussion (L651 – 653), which I don’t think you can state from your work, therefore I would encourage you to consider moving it away from discussing ‘trends’ in the data set, especially when you rightly recommended limitations in the analytical methods/the data.
**Thank you for the comment. We are pleased that the manuscript is now improved. We have deleted the text in lines 651-653 and we have reviewed the discussion to ensure that trends and patterns are made clear and not passed off as significance. We have clarified sections of the discussion to remove ambiguity and ensured that figures are specifically linked to descriptions of the animals’ behaviour.**

Reviewer comment

Please use the space provided to explain your answers to the questions above. You may also include additional comments for the author, including concerns about dual publication, research ethics, or publication ethics. (Please upload your review as an attachment if it exceeds 20,000 characters)

Thank you for taking the time to respond to my review and make subsequent modifications to the manuscript. I think the separation into zoo/wild has made it much easier to follow, and actually has led to some useful comparisons.
**Thank you for the useful and developmental feedback. We appreciate your time and effort in helping shape this manuscript into a more relevant piece of work.**

I have just a few minor comments:
L44: might be worth adding in a sentence about zoos here – i.e. maybe no impact because of the reduced need for animals to show heightened vigilance
**Thank you for the useful edit. We have included this suggestion.**

L291/292: why did you run the test for just some of the sp individually, I think this needs to be all or nothing, unless there is a strong rationale for just selecting some
**Thank you for the comment. This was just the Chi squared for the number of times observed in mixed and single species groups for four example species that were seen in the wild and in the zoo and that may experience a markedly different social environment in the zoo (because of their wild ecology – group number and size, dispersal or distance between individuals) to check for any potential bias or outliers in the species under study. We have included further references to explain why these species were selected.**

Table 3: you have four species here but at L291/292 you just said giraffe and waterbuck
**Edited for clarity in the methods.**

L532/533: I am not sure what this first sentence means
**We have edited this section for clarity.**

L618: there is a word missing before ‘experiencing’, maybe ‘be’?
**Thank you for the correction. We have also edited this section for clarity.**

L641 – 643: I don’t particularly think it is useful to discuss trends, as you don’t want people to take too much from it
**We have re-written this as a call for more research to find out why this trend might occur.**

L264 – 265: is this another trend or is it statistically significant?
**We are not sure what you mean here as these lines are in the methods section? Please can this be clarified.**

L651 – 653: this feels like quite a strong assertion – especially given you have said a few lines before that males spent equal time in mixed and non-mixed groups. I’m concerned this comment might lead to even less interest in bachelor grps, whereas in actual fact we should be doing more than ever to make sure welfare of surplus males is good
**This, and the section in lines 641-643 have been completely re-written. If the reviewer is still unhappy, we can delete this section, but we feel it is worthwhile area of research extension to encourage others to look into.**

L669: remove ‘so’
**Edited**

L670 – 671: this sort of begs the question of why this analysis was done
**We have clarified this section further.**

L689: is this section wild animals, or zoo animals, or both? Needs clarifying
**Edited for clarity.**

L691: what do you mean ‘particularly strong trends’?
**Removed and edited for clarity**
